# Facilitators may represent a new class of regulatory elements

**DOI:** 10.1016/j.gde.2026.102444

**Published:** 2026-04

**Authors:** Mira Kassouf, Alexandra Rampasekova, Doug Higgs

**Affiliations:** 1 Laboratory of Gene Regulation, MRC Weatherall Institute of Molecular Medicine, Radcliffe Department of Medicine, University of Oxford, Oxford, UK; 2 Chinese Academy of Medical Sciences Oxford Institute, University of Oxford, Oxford, UK

## Abstract

Gene expression is controlled by three main cis-regulatory elements: enhancers, promoters, and insulators. When active and bound by transcription factors, co-factors, and architectural proteins, these elements together shape the genome’s structure and function. Although each class has distinct features, the functions of these classes often overlap. Chromatin analyses suggest that the mammalian genome contains over a million cis-regulatory elements. Many are classified as enhancers based on chromatin signatures, and yet they may not act as classical enhancers in traditional assays. However, these enhancer-like elements can augment enhancer activity in their native chromosomal context. We refer to these as ‘facilitators.’ The mechanisms by which facilitators regulate gene expression remain unclear, though two possibilities are proposed. First, facilitators may supply essential factors or raise their concentration to form transcriptional hubs that enhance gene expression. Second, they might help organise chromatin architecture, bringing enhancers and promoters into proximity to increase their interaction. We discuss current evidence suggesting that this subset of uncharacterised cis-acting sequences may constitute a distinct class of regulatory elements that facilitate classical enhancer activity.

**Current Opinion in Genetics & Development** 2026, **97**:102444

This review comes from a themed issue on **Genome Architecture and Expression**

Edited by **Luca Giorgetti** and **Daniel Jost**

For complete overview of the section, please refer to the article collection, “Genome Architecture and Expression (2026)”

https://doi.org/10.1016/j.gde.2026.102444

0959–437X/© 2026 The Authors. Published by Elsevier Ltd. This is an open access article under the CC BY license (http://creativecommons.org/licenses/by/4.0/).

## Introduction

In organisms ranging from fruit flies to humans, gene expression is regulated by three broad classes of cis-regulatory elements: promoters, enhancers, and insulators 1, 2. When such elements are bound by *trans*-acting proteins, they interact with each other 3, 4. These interactions are mediated via different dynamic processes such as thermal diffusion, protein–protein interaction, loop extrusion, liquid–liquid phase separation, alone or in combination, leading to concomitant changes in both genome structure and gene expression 1, 5, 6, 7, 8.

Although the classification of enhancers, promoters, and insulators is firmly established, it has become increasingly clear that, functionally, these elements overlap 4, 9, 10. Enhancers may act as promoters, promoters may act as enhancers, and both may act as insulators 11, 12, 13. Furthermore, an element can behave predominantly as an enhancer in one genomic location and as a promoter when transferred to a different chromosomal region [14]. Following the discoveries of these cis-acting elements, ‘classical assays’ of their function were devised, often involving evaluation in episomal, non-chromatinised assays or random integration into a variety of different, uncharacterised regions of the genome, often using highly mutated cell lines with disordered transcriptional and epigenetic programmes 15, 16, 17. It has emerged that different studies using such approaches often give readouts, which may or may not reflect the true role of *cis*-regulatory elements during development and differentiation in their natural chromosomal environment 18, 19, 20.

More recently, it has been possible to analyse the chromatin signatures of cis-regulatory elements in their natural chromosomal locations to identify them and predict their function. Most cis-regulatory elements are associated with regions of open chromatin, which can be mapped using DNase 1 or via ATAC-seq. Active promoters are located close to transcriptional start sites and, using ChIP-seq, are strongly enriched for H3K4me3 and histone acetylation (H3K27ac). Active enhancers are also enriched for H3K27ac and more strongly enriched for H3K4me1 17, 21, 22. Enhancers also produce small bidirectional eRNAs when intergenic, and both eRNAs and long meRNAs when intragenic 10, 23, 24, 25. Insulators in mammalian cells are identified via their enrichment of CCCTC-binding factor (CTCF). Not surprisingly, when regulatory elements defined by their chromatin signatures are analysed in classical assays, only a proportion of them behave as predicted 19, 20. So, is our model of cis-regulatory elements incomplete, and what are the elements that have the signature of an active enhancer but are not active in classical assays, and how should we characterise them further?

Given the vagaries of the high-throughput assays, the overlapping roles of enhancers, promoters, and insulators, their dependence on chromatin context, and their potentially different roles at different stages of development and differentiation, the ideal experiment for characterising all cis-acting elements is to analyse them in their natural chromosomal context, varying each element one at a time and eventually in informative combinations in appropriate cells throughout development and differentiation. This is now possible using a combination of synthetic biology and genome editing in primary cells and mouse models 26, 27, 28, 29, 30, 31••; however, this is a low-throughput approach, and very few regulatory elements, including enhancer-like elements, have been exhaustively analysed in this way. Nevertheless, this is the most accurate way of characterising cis-regulatory elements and deciphering the fundamental principles linking chromatin architecture and transcriptional regulation. Using this approach to analyse the mammalian alpha-globin locus, we have identified elements with the signature of enhancers that do not act as enhancers in classical assays and yet play an important role in gene regulation [31]. Here, we summarise this potentially new class of cis-regulatory elements, which we termed ‘facilitators,’ and review other recent studies identifying similar regulatory elements in a variety of loci and in different species.

## Identifying and characterising facilitators at the alpha-globin locus

**Figure 1:**
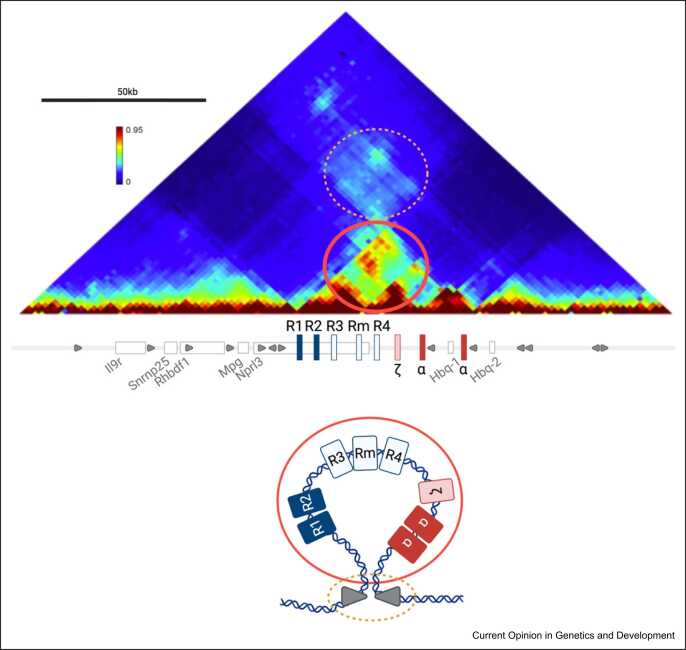
The alpha-globin locus TAD (chr11:32080000-32245000:mm9) and sub-TAD (chr11:32136000-32202000:mm9). Chromatin conformation capture (Tiled-C Capture [37]) contact matrix covering 200kb spanning the mouse alpha-globin cluster with high colour intensity representing increased contact between the alpha-globin major cis-regulatory elements, gene promoters, enhancer-like elements, and CTCF sites, as highlighted by the red circle, embedded within a TAD bounded by CTCF sites (dotted circle). Under the matrix: schematic of the alpha-globin locus depicting promoters of embryonic (ζ) and adult (α) alpha-globin genes (pink and red rectangles respectively), CTCF sites (grey triangles), enhancers R1, R2, facilitators R3, Rm, R4 (dark and light blue rectangles respectively), embedded in the *Nprl3* gene introns (except for R4) and surrounded by other genes as indicated (open rectangles). Below, a depiction of the alpha-globin interaction domain, as in the heatmap red circle, brings the genes and enhancer-like elements into physical proximity, flanked by convergent CTCF sites (dotted circle).

The cis-regulatory elements controlling alpha-globin gene expression have been analysed in considerable detail in mouse embryonic stem cells (mESCs) and in mESCs differentiated into erythroid cells from embryoid bodies [32] and in primary erythroid cells from engineered mice 31••, 33. In mice, this locus contains the duplicated alpha-globin genes (*Hba1* and *Hba2* labelled as α) driven by five erythroid-specific enhancer-like elements (R1, R2, R3, Rm, and R4), all characterised by chromatin marks typical of enhancers (ATAC-seq peak, H3K27ac high, H3K4me1 high, H3K4me3 low). These elements also produce eRNAs and meRNAs only in erythroid cells [23]. This cluster of enhancer-like elements fulfils the definition of a super-enhancer [33]. The super-enhancer and the alpha-genes are flanked by largely oppositely orientated CTCF insulator elements [34] (Figure 1). In erythroid differentiation, using chromosomal conformation capture [35] and super-resolution imaging [36], all five elements, together with the alpha-globin promoters, come into close physical proximity, forming a sub-TAD and simultaneously interacting with each other 37, 38 as alpha-globin expression is switched on to form haemoglobin (Figure 1). This process is thought to be driven, at least in part, by cohesin-mediated loop extrusion [39].

**Figure 2:**
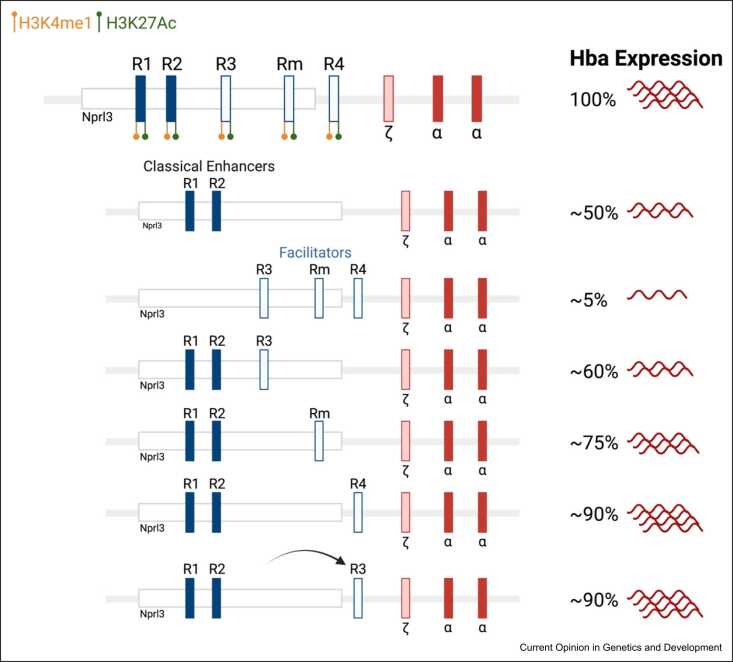
The alpha-globin cluster showing the enhancer elements, the embryonic zeta gene (ζ) and the adult alpha-genes (α). Alpha-globin gene expression (*Hba* expression) is regulated by a super-enhancer constituted by two classical enhancers (R1 and R2) and three facilitators (R3, Rm, and R4), all marked by histone modifications (H3K4me1, H3K27ac, inverted orange and green lollipops). In the absence of facilitators, enhancers R1 and R2 drive only 50% of alpha-globin expression. The facilitators R3, Rm, and R4 have no inherent activation potential; by themselves, they are unable to activate expression. When added to R1, R2, facilitators R3, Rm, and R4 have varying abilities to induce classical enhancer activities (R3: 60%, Rm: 75%, R4: 90%), with promoter-proximal R4 exerting the strongest activation effect. When the weakest facilitator R3 is placed in the R4 position, its activity is boosted, showing a position-dependent function of facilitators.

In an initial study, deletion of each enhancer-like element showed that most of the enhancer activity was provided by two classical enhancers (R1 and R2). R3, Rm, and R4 had no enhancer activity in the context of the otherwise intact locus or in any of the standard enhancer assays [33]. Based on these experiments, we initially concluded that the enhancer-like elements interacted in an additive manner and the role of R3, Rm, and R4, if any, was not clear [33]. In a subsequent set of experiments, we created an enhancerless locus and added back the enhancer-like elements individually and in informative combinations. This showed that the classical enhancers (R1 and R2) on their own or in combination expressed relatively high, albeit suboptimal levels of alpha-globin RNA (∼50%) in the absence of R3, Rm, and R4. However, the addition of these enhancer-like elements restored full expression. Importantly, the degree of restoration depended on the positions occupied by the enhancer-like elements (Figure 2). We concluded that although these elements have little or no intrinsic enhancer activity, they nevertheless increase the activity of classical enhancers such as R1 and R2. Consequently, we referred to these elements as facilitators [31].

Based on these findings, we define a facilitator as a cis-acting regulatory element with the classical signature of an enhancer (DNase, ATAC-Seq, H3K27ac, H3K4me1, eRNAs) but no enhancer activity in reporter assays. When tested in its natural genomic position, a facilitator drives either no or very weak expression of its target gene in the absence of a classical enhancer. Nevertheless, when in combination, a facilitator significantly increases the effect of the classical enhancer.

## Other observations consistent with the presence of facilitators

Recent studies have provided evidence that, similar to the alpha-globin locus, clusters of enhancers (super-enhancers) are often composed of multiple functionally distinct elements, some of which may act as facilitators.

**Figure 3:**
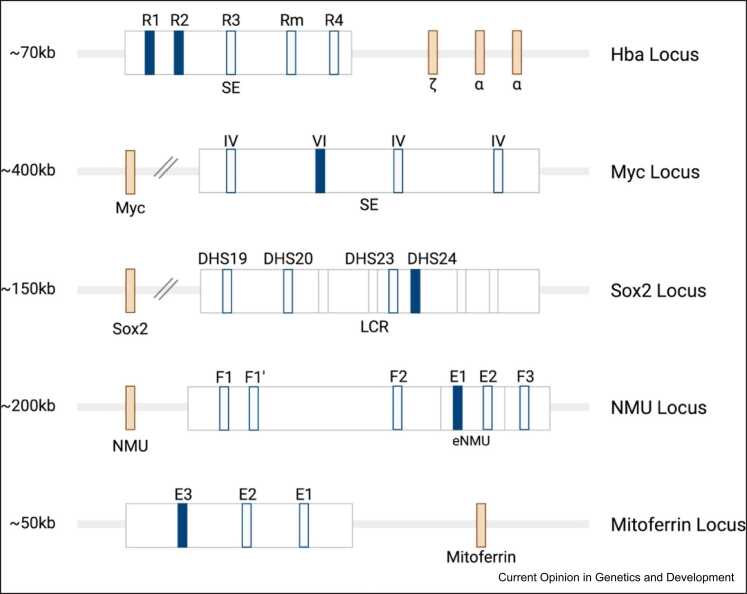
Super-enhancer constituent elements of representative loci with the gene marked in orange rectangles, classical enhancers in dark blue rectangles, and facilitator-like elements in light blue rectangles.

Sahu et al. [40] noted that some frequently occurring cis-acting elements with the signatures of enhancers have no intrinsic enhancer activity in the STARR-seq assay (an example of a classical enhancer assay), and they suggested that these elements may boost the expression of closely linked enhancers. They referred to such elements as ‘chromatin-dependent enhancers’ [40]. They found that super-enhancers, such as one of the *MYC* super-enhancers (Figure 3), typically consist of arrays of elements comprising one or more classical enhancers linked to chromatin-dependent enhancers. However, these elements were not tested directly within the context of the *MYC* locus.

Recent analysis of the mouse *Sox2* locus has shown that similar cis-elements, here called ‘context-dependent enhancers,’ may also exist at this locus [41]. Expression of *Sox2* is controlled by a highly complex landscape containing 28 *cis*-acting regulatory elements (Figure 3), including a key classical enhancer (DHS24), which scores highly in a STARR-seq assay, and when deleted from the endogenous locus, reduces expression of *Sox2* to ∼45% of its normal expression level. When all three context-dependent enhancers (DHS19, DHS20, and DHS23) are deleted together, they cause only a minor degree (∼10%) of downregulation, but when, for example, DHS23 is deleted in combination with DHS24, expression of *Sox2* is more severely reduced. From this and further studies analysing multiple combinations of cis-regulatory elements from mESC regulator loci engineered into the *Sox2* locus control region (LCR), the authors concluded that while context-dependent enhancers cannot work alone, they may double the activity of neighbouring enhancers [42]. The complexity of the locus makes interpretation difficult, but these observations are consistent with context-dependent enhancers acting as facilitators.

Using mESCs, the laboratory of Christa Buecker engineered a testbed for enhancer cooperativity and function within the mouse beta-globin locus by inserting landing pads located 1.5 kb, 25 kb, and 75kb upstream of a minimal TK promoter driving expression of mCherry [29]. Since the beta-globin locus is inactive in mESCs, this was considered to be a neutral locus. In this system, they found that all enhancers show reduced ability to activate a promoter with increasing distance. Furthermore, they found that combinations of weak and strong enhancers significantly increase expression levels when the weak enhancer is placed between the strong enhancer and the promoter. This is very reminiscent of the situation seen at the alpha globin cluster in erythroid cells, where the facilitators lie between the classical enhancers and their target promoters for the alpha genes.

Recent analysis of the *NMU* locus has provided similar conclusions [43]. This locus contains the NMU gene, a classical enhancer (eNMU-e1), and five putative facilitator elements (eNMU-e2, F1, F′, F2, and F3) within a region of ∼98 kb (Figure 3) 43, 44, 45. Like alpha-globin, expression of *NMU* is greatly upregulated during erythroid differentiation. In the absence of the enhancer, these elements, also referred to as facilitators, fail to activate expression of *NMU*. From changes in chromatin accessibility and epigenetic modifications during erythropoiesis, the authors suggest that the *NMU* facilitators play an important role in complementing the classical enhancer to regulate gene expression in erythroid differentiation, but, at present, expression of the *NMU* enhancer has not been tested in the absence of all five of the proposed facilitators.

Similar changes in the function of enhancers during erythroid differentiation were made when studying the *Mitoferrin* gene in the mouse [46]. The super-enhancer presents a functional hierarchy within its three constituents (E1, E2, E3); upon deletion of the three elements individually or in combination, E3 has the most significant effect on mouse Mitoferrin expression in differentiated erythroid cells and is considered the critical enhancer (E3), but cooperates with E1 and E2 elements for optimal enhancer activity (Figure 3). Further examples of putative facilitators have been reported at super-enhancers controlling the human *EphA2* locus [47], the mouse *miR-290-295* locus [48], and the mouse *Wap* locus [49].

## ‘Weak enhancers’ may act as facilitators

‘Weak enhancers’ are genomic *cis*-regulatory elements that show lower intrinsic enhancer activity than classical or ‘strong’ enhancers. However, this definition involves somewhat poorly defined subjective criteria: lower reporter activity, lower levels of active chromatin marks (ATAC peaks, H3K27ac), lower eRNA production, and fewer bound lineage-specific transcription factors (TFs) and co-factors (Mediator, p300CBP, BRD4). Despite the pitfalls in quantifying these assays, weak enhancers defined in these ways may contribute to gene regulation by fine-tuning or providing redundancy and/or robustness to gene expression.

Recently, Kribelbauer-Swietek et al. identified a set of enhancer-like elements whose contribution to gene expression can be altered by naturally occurring single nucleotide polymorphisms [50]. Driven by features associated with their corresponding TF binding sites (episomal versus endogenous enhancer activities), they then defined chromatin modules (CMs) in which two or more of these TFs act coordinately. They considered regulatory element communication to occur whenever a functional SNP in one element changed the epigenetic signature and/or function of the other. In this study, they distinguished between ‘lead’ elements, which initiate this process, and ‘dependent’ elements, which respond to changes in the lead element. Importantly, in classical assays, lead elements behaved as strong enhancers, and dependent enhancer-like elements behaved as weak enhancers. In addition, they recruited different classes of TFs. The authors concluded that CMs show an internal hierarchy: lead enhancers are classical enhancers, whereas dependent enhancer-like elements appear to play a role in communication between enhancers and promoters, as proposed for facilitators.

A study on the *Fgf5* locus [51], which is regulated by five enhancer-like elements (poised promoter proximal enhancer PE, and E1-E4), reports similar observations. All five *Fgf5* enhancer-like elements have low intrinsic enhancer activity in reporter assays, while deletion of individual elements in their natural context affects *Fgf5* gene expression upon differentiation to varying degrees, except E4. The authors conclude that each element by itself has low enhancer activity, and only when all of these ‘weak’ enhancers are combined in the correct genomic context can they strongly activate gene expression. In this case, there appears to be no classical enhancer; rather, there are four weak enhancers working in a super-additive manner, in different combinations, during differentiation of mESCs to epiblast-like cells. The intronic element closest to the promoter (PE), a noncanonical enhancer, is described as an ‘amplifier.’ Whether this locus organisation fulfils an enhancer–facilitator arrangement remains to be confirmed.

Finally, attention has recently been refocused on promoter-proximal elements, which have been shown to determine the degree to which specific pairs of enhancers and promoters might interact. The authors suggest that within multipartite regulatory domains, the promoter-proximal region fulfils ‘a facilitator-like function’ that filters and transmits signal from distal enhancers, ultimately conferring enhancer–promoter compatibility 52, 53.

## Other key regulatory elements with no intrinsic enhancer activity: insulators, tethering elements, and range-extender elements

In mammals, the predominant insulator protein is CTCF, which is thought to play a key role in delimiting chromatin loop extrusion by cohesin, creating TADs and sub-TADs, and thereby increasing or decreasing interactions between specific enhancers and promoters [54]. By contrast, although present in *Drosophila,* cohesin does not drive robust CTCF-anchored loop extrusion as in mammals. Instead, genome organisation and enhancer–promoter interaction in flies appears to be shaped more by protein–protein interactions driven by a variety of insulator proteins, including BEAF-32, CP190, and Su(Hw) [55]. These proteins interact with other proteins bound at or near regulatory elements in the locus and pair with each other. As in mammals, insulators in *Drosophila* may increase or decrease interactions between enhancers and promoters 54, 55, 56.

Recently, a distinct class of approximately 300–500 base-pair *cis*-acting regulatory sequences known as tethering elements (TEs) has been identified in *Drosophila* [57]. TEs do not possess intrinsic enhancer activities when attached to naive reporter genes and do not bear the classical chromatin signatures of enhancers or promoters. However, these elements mediate highly specific long-range genomic interactions, forming enhancer–promoter or promoter–promoter loops 56, 58. They often contain repeated sequences. Functionally, TEs act via physical interactions between elements that most commonly bind the GAGA-associated factor or components of the Polycomb (Pc) complex [57]. The potential connection between TEs and the Pc complex is interesting, given the association of Pc with CpG islands in mammalian cells [59]. Notably, a subgroup of CpG islands referred to as orphan CpG islands has been shown to possess tethering activities. These activities play a crucial role in facilitating both the physical and functional communication between enhancers and genes located at a distance. Orphan CpG islands do not lie close to promoters; they lack intrinsic enhancer activity but are thought to promote long-range interactions between enhancers and CpG-rich developmental promoters [59]. Of note, although TEs act over medium to long-range distances, some have been shown to bring together cis-elements that lie megabases apart in the genome [52]. The presence of TEs in mammals is less clear. Bower et al. have recently reported a prototypical range extender (REX) element at the *Sall1* locus that confers long-distance regulatory activity on a closely linked classical enhancer [60]. Again, the REX element has no intrinsic enhancer activity. However, addition of the REX element to other short- and mid-range limb enhancers substantially increases their genomic interaction range. The REX element and related elements contain highly conserved homeodomain motifs that are critical for their activity [60].

In the context of the beta-globin gene locus [61] and other loci [62], it has clearly been shown that elements binding LDB1, recruited via GATA1, may behave like TEs. The *Drosophila* homologue of Ldb1 is Chip, which is required for long-range enhancer–promoter associations at the cut locus [63]. Of interest, Chip is recruited to cut via Pannier, a zinc finger protein of the GATA family. In the recently reported data from Gerd Blobel’s lab [64], it was shown that LDB1 binds all elements within the alpha-globin super-enhancer but not the alpha-globin promoters, suggesting that Ldb1 may be important in bringing the enhancer-like elements into juxtaposition.

In a study of the *Sox9* locus, Chen et al. reported the presence of two elements important for mediating ultra–long-range interactions (>1 Mb) between the enhancers and promoters [65]. These elements are associated with the presence of interaction stripes in imaging-based chromosome conformation maps and were referred to as stripe-associated structural elements (SSEs). When deleted, these elements had a significant effect on Sox9 gene expression despite not having any intrinsic enhancer activity. SSEs bind CTCF, but the authors conclude that instead of promoting insulation or directionality, they facilitate the function of distal enhancer clusters in the *SOX9* locus by enabling the formation of a multiloop structure and compaction of the entire domain. Given the definition of facilitators set out here, it seems likely that some facilitators will overlap in structure and/or function with insulators, TEs, and REX elements, as described in this section.

## Mechanisms of action

**Figure 4:**
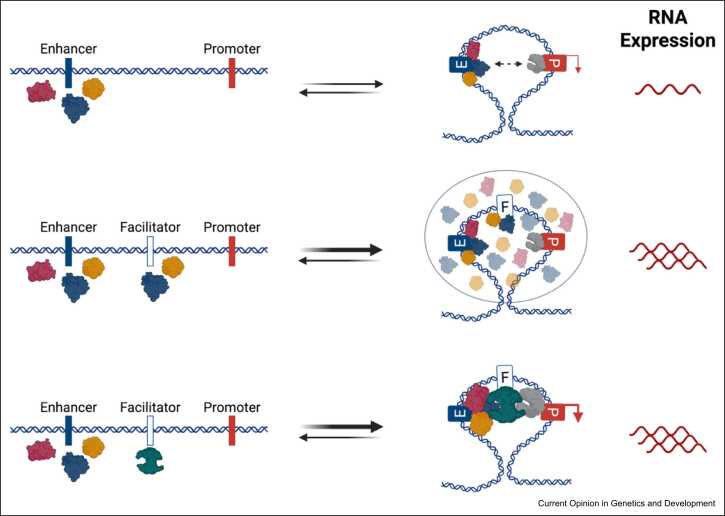
Proposed models of interaction between enhancers, facilitators, and the alpha-globin genes. Increased alpha-globin expression (RNA Expression, red curvy lines) is concomitant with increased interaction between enhancers (E) and promoters (P) (top), either via higher concentration of transcription factors and co-factors within a transcriptional hub (middle) or an established physical proximity facilitated by architectural proteins bound to facilitators (F) (bottom), amongst other proposed mechanisms.

We propose that facilitators might contribute to enhancer-driven gene expression in at least two novel ways. First, they may increase the repertoire and/or concentration of proteins required to form a transcriptional hub (Figure 4) as proposed for the weak ‘dependent’ enhancers described above [50]. Second, they may play an architectural role in bringing regulatory elements into closer proximity and stabilising their interaction (Figure 4), as shown for TEs and some weak enhancers [29].

Some elements may contribute to both roles. Recent experiments at the *Sox2* locus have shown that an element called SSR2 can behave as a weak enhancer. However, it also appears to play a role in bringing the super-enhancer and the *Sox2* promoter together [66]. When the SSR2 element is in place, *Sox2* expression does not depend on loop extrusion. However, when SSR2 is deleted, long-range enhancer–promoter interaction and expression of *Sox2* now become dependent on loop extrusion. This suggests that SSR2, a weak enhancer, plays a role in bringing the enhancer and promoter into proximity to each other in a manner that is redundant with loop extrusion.

## Conclusions

To date, from a very limited number of observations, we can define facilitators as elements that, to a variable extent, share the signatures of enhancers, promoters, and insulators, and yet do not function in the classical ways ascribed to these elements. Nevertheless, *in vivo*, facilitators significantly increase the activity of a linked endogenous classical enhancer in a position-dependent manner. As with all classes of regulatory elements, facilitators will overlap with other regulatory elements, but their separate classification may focus further research on less well-studied regulatory elements that contribute in novel ways to enhancer-driven gene expression.

At present, there are too few observations to make a clear distinction between weak enhancers, architectural elements, and facilitators. Based on the reconstruction of the alpha-globin locus and other observations described in this review, a working definition would describe facilitators as elements often within a cluster of enhancer-like elements, which, at their natural position, in the absence of a classical enhancer, drive little or no expression, whereas their removal from the cluster significantly reduces enhancer-driven expression. To take this further, finding a specific chromatin signature for facilitators would allow their genome-wide identification in various cellular and developmental contexts and establish a functional assay to help distinguish them from other regulatory elements.

## Declaration of Competing Interest

The authors declare no conflict of interest.

## Data Availability

No data were used for the research described in the article.

## References

[1] Yang J.H., Hansen A.S. (2024). Enhancer selectivity in space and time: from enhancer–promoter interactions to promoter activation. Nat Rev Mol Cell Biol.

[2] Furlong E.E.M., Levine M. (2018). Developmental enhancers and chromosome topology. Science.

[3] Spitz F., Furlong E.E.M. (2012). Transcription factors: from enhancer binding to developmental control. Nat Rev Genet.

[4] Zabidi M.A., Stark A. (2016). Regulatory enhancer–core-promoter communication via transcription factors and cofactors. Trends Genet.

[5] Dekker J., Mirny L.A. (2024). The chromosome folding problem and how cells solve it. Cell.

[6] Karpinska M.A., Oudelaar A.M. (2023). The role of loop extrusion in enhancer-mediated gene activation. Curr Opin Genet Dev.

[7] Galouzis C.C., Furlong E.E.M. (2022). Regulating specificity in enhancer–promoter communication. Curr Opin Cell Biol.

[8] Lim B., Levine M.S. (2021). Enhancer-promoter communication: hubs or loops?. Curr Opin Genet Dev.

[9] Kim T.-K., Shiekhattar R. (2015). Architectural and functional commonalities between enhancers and promoters. Cell.

[10] Adelman A.F., Adelman K. (2020). Evaluating enhancer function and transcription. Annu Rev Biochem.

[11] Raab J.R., Kamakaka R.T. (2010). Insulators and promoters: closer than we think. Nat Rev Genet.

[12] Andersson R., Sandelin A. (2020). Determinants of enhancer and promoter activities of regulatory elements. Nat Rev Genet.

[13] Harrold C.L., Gosden M.E., Hanssen L.L.P., Stolper R.J., Downes D.J., Telenius J.M., Biggs D., Preece C., Alghadban S., Sharpe J.A. (2020). A functional overlap between actively transcribed genes and chromatin boundary elements. Biorxiv.

[14] Georgiades E., Harrold C., Roberts N., Kassouf M., Riva S.G., Sanders E., Downes D., Francis H.S., Blayney J., Oudelaar A.M. (2025). Active regulatory elements recruit cohesin to establish cell specific chromatin domains. Sci Rep.

[15] Mannion B.J., Tran S., Plajzer-Frick I., Novak C.S., Afzal V., Akiyama J.A., Sospedra-Arrufat I., Barton S., Beckman E., Garvin T.H. (2025). Uncovering hidden enhancers through unbiased in vivo testing. Nat Commun.

[16] Gorkin D.U., Barozzi I., Zhao Y., Zhang Y., Huang H., Lee A.Y., Li B., Chiou J., Wildberg A., Ding B. (2020). An atlas of dynamic chromatin landscapes in mouse fetal development. Nature.

[17] Consortium T.E.P., Abascal F., Acosta R., Addleman N.J., Adrian J., Afzal V., Ai R., Aken B., Akiyama J.A., Jammal O.A. (2020). Expanded encyclopaedias of DNA elements in the human and mouse genomes. Nature.

[18] Inoue F., Kircher M., Martin B., Cooper G.M., Witten D.M., McManus M.T., Ahituv N., Shendure J. (2017). A systematic comparison reveals substantial differences in chromosomal versus episomal encoding of enhancer activity. Genome Res.

[19] Barakat T.S., Halbritter F., Zhang M., Rendeiro A.F., Perenthaler E., Bock C., Chambers I. (2018). Functional dissection of the enhancer repertoire in human embryonic stem cells. Cell Stem Cell.

[20] Catarino R.R., Stark A. (2018). Assessing sufficiency and necessity of enhancer activities for gene expression and the mechanisms of transcription activation. Genes Dev.

[21] Heintzman N.D., Stuart R.K., Hon G., Fu Y., Ching C.W., Hawkins R.D., Barrera L.O., Calcar S.V., Qu C., Ching K.A. (2007). Distinct and predictive chromatin signatures of transcriptional promoters and enhancers in the human genome. Nat Genet.

[22] Heintzman N.D., Hon G.C., Hawkins R.D., Kheradpour P., Stark A., Harp L.F., Ye Z., Lee L.K., Stuart R.K., Ching C.W. (2009). Histone modifications at human enhancers reflect global cell-type-specific gene expression. Nature.

[23] Kowalczyk M.S., Hughes J.R., Garrick D., Lynch M.D., Sharpe J.A., Sloane-Stanley J.A., McGowan S.J., De Gobbi M., Hosseini M., Vernimmen D. (2012). Intragenic enhancers act as alternative promoters. Mol Cell.

[24] Kim T.-K., Hemberg M., Gray J.M., Costa A.M., Bear D.M., Wu J., Harmin D.A., Laptewicz M., Barbara-Haley K., Kuersten S. (2010). Widespread transcription at neuronal activity-regulated enhancers. Nature.

[25] Santa F.D., Barozzi I., Mietton F., Ghisletti S., Polletti S., Tusi B.K., Muller H., Ragoussis J., Wei C.-L., Natoli G. (2010). A large fraction of extragenic RNA Pol II transcription sites overlap enhancers. PLoS Biol.

[26] Brosh R., Laurent J.M., Ordoñez R., Huang E., Hogan M.S., Hitchcock A.M., Mitchell L.A., Pinglay S., Cadley J.A., Luther R.D. (2021). A versatile platform for locus-scale genome rewriting and verification. Proc Natl Acad Sci USA.

[27] Mitchell L.A., McCulloch L.H., Pinglay S., Berger H., Bosco N., Brosh R., Bulajić M., Huang E., Hogan M.S., Martin J.A. (2021). De novo assembly and delivery to mouse cells of a 101 kb functional human gene. Genetics.

[28] Boeke J.D., Church G., Hessel A., Kelley N.J., Arkin A., Cai Y., Carlson R., Chakravarti A., Cornish V.W., Holt L. (2016). Genome engineering. The Genome Project-Write. Science.

[29] Thomas H.F., Feng S., Haslhofer F., Huber M., Gallardo M.G., Loubiere V., Vanina D., Pitasi M., Stark A., Buecker C. (2025). Enhancer cooperativity can compensate for loss of activity over large genomic distances. Mol Cell.

[30] Camellato B.R., Brosh R., Ashe H.J., Maurano M.T., Boeke J.D. (2024). Synthetic reversed sequences reveal default genomic states. Nature.

[31••] Blayney J.W., Francis H., Rampasekova A., Camellato B., Mitchell L., Stolper R., Cornell L., Babbs C., Boeke J.D., Higgs D.R. (2023). Super-enhancers include classical enhancers and facilitators to fully activate gene expression. Cell.

[32] Francis H.S., Harold C.L., Beagrie R.A., King A.J., Gosden M.E., Blayney J.W., Jeziorska D.M., Babbs C., Higgs D.R., Kassouf M.T. (2022). Scalable in vitro production of defined mouse erythroblasts. PLoS One.

[33] Hay D., Hughes J.R., Babbs C., Davies J.O.J., Graham B.J., Hanssen L.L.P., Kassouf M.T., Oudelaar A.M., Sharpe J.A., Suciu M.C. (2016). Genetic dissection of the α-globin super-enhancer in vivo. Nat Genet.

[34] Hanssen L.L.P., Kassouf M.T., Oudelaar A.M., Biggs D., Preece C., Downes D.J., Gosden M., Sharpe J.A., Sloane-Stanley J.A., Hughes J.R. (2017). Tissue-specific CTCF/Cohesin-mediated chromatin architecture delimits enhancer interactions and function in vivo. Nat Cell Biol.

[35] Davies J.O.J., Telenius J.M., McGowan S., Roberts N.A., Taylor S., Higgs D.R., Hughes J.R. (2016). Multiplexed analysis of chromosome conformation at vastly improved sensitivity. Nat Methods.

[36] Brown J.M., Roberts N.A., Graham B., Waithe D., Lagerholm C., Telenius J.M., Ornellas S.D., Oudelaar A.M., Scott C., Szczerbal I. (2018). A tissue-specific self-interacting chromatin domain forms independently of enhancer-promoter interactions. Nat Commun.

[37] Oudelaar A.M., Beagrie R.A., Gosden M., de Ornellas S., Georgiades E., Kerry J., Hidalgo D., Carrelha J., Shivalingam A., El-Sagheer A.H. (2020). Dynamics of the 4D genome during in vivo lineage specification and differentiation. Nat Commun.

[38] Hua P., Badat M., Hanssen L.L.P., Hentges L.D., Crump N., Downes D.J., Jeziorska D.M., Oudelaar A.M., Schwessinger R., Taylor S. (2021). Defining genome architecture at base-pair resolution. Nature.

[39] Stolper R.J., Tsang F.H., Georgiades E., Hansen L.L.P., Downes D.J., Harrold C.L., Hughes J.R., Beagrie R.A., Davies B., Kassouf M.T. (2023). Loop extrusion by cohesin plays a key role in enhancer-activated gene expression during differentiation. bioRxiv.

[40] Sahu B., Hartonen T., Pihlajamaa P., Wei B., Dave K., Zhu F., Kaasinen E., Lidschreiber K., Lidschreiber M., Daub C.O. (2022). Sequence determinants of human gene regulatory elements. Nat Genet.

[41•] Brosh R., Coelho C., Ribeiro-dos-Santos A.M., Ellis G., Hogan M.S., Ashe H.J., Somogyi N., Ordoñez R., Luther R.D., Huang E. (2023). Synthetic regulatory genomics uncovers enhancer context dependence at the Sox2 locus. Mol Cell.

[42] Ordoñez R., Ribeiro-dos-Santos A.M., McLoughlin C., Ellis G., Ashe H.J., Zufiaurre N.B., Brosh R., Majewski M., Camellato B., Boeke J.D. (2025). Synthetic genomic dissection of enhancer context sensitivity and synergy. bioRxiv.

[43] Zhou Z., Li A., Zhang J., Yu H., Ozer A., Lis J.T. (2025). Robust regulatory interplay of enhancers, facilitators, and promoters in a native chromatin context. bioRxiv.

[44] Gasperini M., Hill A.J., McFaline-Figueroa J.L., Martin B., Kim S., Zhang M.D., Jackson D., Leith A., Schreiber J., Noble W.S. (2019). A genome-wide framework for mapping gene regulation via cellular genetic screens. Cell.

[45] Reilly S.K., Gosai S.J., Gutierrez A., Mackay-Smith A., Ulirsch J.C., Kanai M., Mouri K., Berenzy D., Kales S., Butler G.M. (2021). Direct characterization of cis-regulatory elements and functional dissection of complex genetic associations using HCR–FlowFISH. Nat Genet.

[46] Huang J., Liu X., Li D., Shao Z., Cao H., Zhang Y., Trompouki E., Bowman T.V., Zon L.I., Yuan G.-C. (2016). Dynamic control of enhancer repertoires drives lineage and stage-specific transcription during hematopoiesis. Dev Cell.

[47] Cui S., Wu Q., Liu M., Su M., Liu S., Shao L., Han X., He H. (2021). EphA2 super-enhancer promotes tumor progression by recruiting FOSL2 and TCF7L2 to activate the target gene EphA2. Cell Death Dis.

[48] Hnisz D., Schuijers J., Lin C.Y., Weintraub A.S., Abraham B.J., Lee T.I., Bradner J.E., Young R.A. (2015). Convergence of developmental and oncogenic signaling pathways at transcriptional super-enhancers. Mol Cell.

[49] Shin H.Y., Willi M., Yoo K.H., Zeng X., Wang C., Metser G., Hennighausen L. (2016). Hierarchy within the mammary STAT5-driven Wap super-enhancer. Nat Genet.

[50••] Kribelbauer-Swietek J.F., Pushkarev O., Gardeux V., Faltejskova K., Russeil J., Mierlo G. van, Deplancke B. (2024). Context transcription factors establish cooperative environments and mediate enhancer communication. Nat Genet.

[51] Thomas H.F., Kotova E., Jayaram S., Pilz A., Romeike M., Lackner A., Penz T., Bock C., Leeb M., Halbritter F. (2021). Temporal dissection of an enhancer cluster reveals distinct temporal and functional contributions of individual elements. Mol Cell.

[52] Majchrzycka B., Mundlos S., Krebs A.R., Ibrahim D.M. (2025). Enhancer-promoter compatibility is mediated by the promoter-proximal region. bioRxiv.

[53] Masoura M., Balasubramanian D., Moretti C., Lison M., Tarayre H., Lajoignie D., Cretet-Rodeschini C., Cadet L., Mendes J., Vincent S. (2025). Promoter-proximal gatekeepers restrict pleiotropic enhancer inputs to achieve tissue specificity. bioRxiv.

[54] Beagan J.A., Phillips-Cremins J.E. (2020). On the existence and functionality of topologically associating domains. Nat Genet.

[55] Phillips-Cremins J.E., Corces V.G. (2013). Chromatin insulators: linking genome organization to cellular function. Mol Cell.

[56] Batut P.J., Bing X.Y., Sisco Z., Raimundo J., Levo M., Levine M.S. (2022). Genome organization controls transcriptional dynamics during development. Science.

[57] Li X., Levine M. (2024). What are tethering elements?. Curr Opin Genet Dev.

[58] Levo M., Raimundo J., Bing X.Y., Sisco Z., Batut P.J., Ryabichko S., Gregor T., Levine M.S. (2022). Transcriptional coupling of distant regulatory genes in living embryos. Nature.

[59] Pachano T., Sánchez-Gaya V., Ealo T., Mariner-Faulí M., Bleckwehl T., Asenjo H.G., Respuela P., Cruz-Molina S., Martín M.M.-S., Haro E. (2021). Orphan CpG islands amplify poised enhancer regulatory activity and determine target gene responsiveness. Nat Genet.

[60••] Bower G., Hollingsworth E.W., Jacinto S.H., Alcantara J.A., Clock B., Cao K., Liu M., Dziulko A., Alcaina-Caro A., Xu Q. (2025). Range extender mediates long-distance enhancer activity. Nature.

[61] Krivega I., Dean A. (2017). LDB1-mediated enhancer looping can be established independent of mediator and cohesin. Nucleic Acids Res.

[62] Kwon H., Kim J., Zhou L., Dean A. (2025). LDB1 regulates gene expression and chromatin structure in pluripotency and lineage differentiation. bioRxiv.

[63] Morcillo P., Rosen C., Baylies M.K., Dorsett D. (1997). Chip, a widely expressed chromosomal protein required for segmentation and activity of a remote wing margin enhancer in Drosophila. Genes Dev.

[64] Aboreden N.G., Lam J.C., Goel V.Y., Wang S., Wang X., Midla S.C., Quijano A., Keller C.A., Giardine B.M., Hardison R.C. (2025). LDB1 establishes multi-enhancer networks to regulate gene expression. Mol Cell.

[65] Chen L.-F., Long H.K., Park M., Swigut T., Boettiger A.N., Wysocka J. (2023). Structural elements promote architectural stripe formation and facilitate ultra-long-range gene regulation at a human disease locus. Mol Cell.

[66••] Hansen K.L., Adachi A.S., Braccioli L., Kadvani S., Boileau R.M., Martinovic M., Pokorny B., Shah R., Anderson E.C., Zhang K. (2025). Synergy between regulatory elements can render cohesin dispensable for distal enhancer function. Science.

